# Hippocampal Neurogenesis, Cognitive Deficits and Affective Disorder in Huntington's Disease

**DOI:** 10.1155/2012/874387

**Published:** 2012-06-27

**Authors:** Mark I. Ransome, Thibault Renoir, Anthony J. Hannan

**Affiliations:** ^1^Florey Neuroscience Institutes, Melbourne Brain Centre, University of Melbourne, Melbourne, VIC 3010, Australia; ^2^Department of Anatomy and Cell Biology, University of Melbourne, Melbourne, VIC 3010, Australia

## Abstract

Huntington's disease (HD) is a neurodegenerative disorder caused by a tandem repeat expansion encoding a polyglutamine tract in the huntingtin protein. HD involves progressive psychiatric, cognitive, and motor symptoms, the selective pathogenesis of which remains to be mechanistically elucidated. There are a range of different brain regions, including the cerebral cortex and striatum, known to be affected in HD, with evidence for hippocampal dysfunction accumulating in recent years. In this review we will focus on hippocampal abnormalities, in particular, deficits of adult neurogenesis. We will discuss potential molecular mechanisms mediating disrupted hippocampal neurogenesis, and how this deficit of cellular plasticity may in turn contribute to specific cognitive and affective symptoms that are prominent in HD. The generation of transgenic animal models of HD has greatly facilitated our understanding of disease mechanisms at molecular, cellular, and systems levels. Transgenic HD mice have been found to show progressive behavioral changes, including affective, cognitive, and motor abnormalities. The discovery, in multiple transgenic lines of HD mice, that adult hippocampal neurogenesis and synaptic plasticity is disrupted, may help explain specific aspects of cognitive and affective dysfunction. Furthermore, these mouse models have provided insight into potential molecular mediators of adult neurogenesis deficits, such as disrupted serotonergic and neurotrophin signaling. Finally, a number of environmental and pharmacological interventions which are known to enhance adult hippocampal neurogenesis have been found to have beneficial affective and cognitive effects in mouse models, suggesting common molecular targets which may have therapeutic utility for HD and related diseases.

## 1. Introduction 

Toward the close of the nineteenth century, the Neuron Doctrine emerged as a fundamental theory of the nervous system [1]. A basic tenet of the Neuron Doctrine was that specialized cells—neurons—formed the elementary structural and functional units of the nervous system. Embedded within the formulation and refinement of the Neuron Doctrine was born the concept of neuronal plasticity or structural modifiability of the brain [2]. Arguably, and perhaps unwittingly, the developing theory of neural plasticity was linked with functional roles in learning and memory. However, the ability of the brain to modify its connections was tempered by the overriding edict that the adult neuronal population was stable. In other words, production of new neurons—neurogenesis—was complete prior to brain maturation. Thus another basic tenet of the Neuron Doctrine was that the adult mammalian brain was devoid of ongoing neurogenesis. This view of adult neurogenesis pervaded neuroscience for a substantial portion of the twentieth century, holding sway in spite of early contradictory evidence [3, 4]. With technical advancements and overwhelming evidence, adult neurogenesis in the mammalian brain became a widely accepted concept by the close of the twentieth century [5]. Addition of new neurons to established circuitry of the adult brain affords a novel form of neuronal plasticity beyond the structural and synaptic plasticity of existing neurons. Adult neurogenesis is considered to play an etiological role in, and provide a therapeutic target for, many neurodegenerative conditions [6]. The potential of manipulating adult neurogenesis to treat the cognitive deficits associated with Huntington's disease has been recently covered in detail [7]. This review will focus on an emerging association between adult neurogenesis and hippocampal dysfunction and its relationship to affective and cognitive symptoms in Huntington's disease. 

## 2. Hippocampal Neurogenesis and Huntington's Disease 

The hippocampus is a key structural element of the limbic system and has been intensively investigated as a mediator of learning and memory [8]. Beyond cognition, however, impaired hippocampal neuronal plasticity is thought to be a significant neural substrate underlying the development of depressive behavior [9, 10]. Major depressive disorder encompasses cognitive impairment and both impaired cognition and depression feature in the spectrum of symptoms experienced by HD patients [11]. A significant step toward the general acceptance of adult neurogenesis was the generation of evidence for neuron production in the adult human hippocampus [12]. Since this study, there has been, and continues to be, considerable debate regarding the functional significance of hippocampal neurogenesis in human health and disease [13]. Hypotheses have been put forward that posit (1) impaired neurogenesis elicits the development of depression and (2) that efficacy of antidepressants relies on intact hippocampal neurogenesis [14–16]. Refinement (or refutation) of the “neurogenesis hypothesis” of depression will have an impact not only on our understanding of pathogenic mechanisms but also on the development of new treatments. Recent studies using transgenic HD mice may further inform the neurogenesis hypothesis of depression and provide preclinical direction for more effective HD treatments. 

Adult hippocampal neurogenesis (AHN) is a highly regulated multistep process involving proliferation of neuronal stem/precursor cells, neuronal differentiation and migration, and finally neuronal maturation and integration into the existing hippocampal circuitry [17]. A diversity of factors targets these regulatory steps to affect the net rate of AHN. A significant foundation for the neurogenesis hypothesis of depression arises from data that shows neurotransmitter systems involved in the development and treatment of depression also regulate levels of AHN [14]. Various transgenic rodent models of HD have been found to exhibit affective and cognitive abnormalities reflecting clinical data in HD patients. For example, R6/1 and R6/2 transgenic lines of HD mice have behavioral deficits that include impaired hippocampal-dependent spatial cognition [18–20]. However, depressive-like behavior also manifests in R6/1 HD mice prior to cognitive and motor symptoms [21, 22]. 

Serotonin is a key neurotransmitter system involved in the etiology and treatment of depressive disorders [23, 24]. Selective serotonin uptake inhibitors (SSRIs), such as fluoxetine (Prozac) and sertraline continue to be a core pharmacological intervention for major depressive disorder [23, 24]. A significant mechanism of action of the antidepressant effects of SSRIs involves potentiation of serotonergic neurotransmission through inhibition of synaptic clearance. Reflecting the incidence of HD patient depression, depressive-like behavior manifests in premotor symptomatic R6/1 HD mice [21, 25]. Early analysis of R6/1 HD mice showed a reduction in hippocampal stem/precursor cell proliferation leading to a net reduction in AHN [26]. Further studies in R6/1 [25, 27, 28] and R6/2 [29–31] lines of mice have extended our understanding of the AHN deficit. Could the impaired AHN in R6/1 HD mice cause the development of the depressive-like phenotype? Administration of fluoxetine to R6/1 HD mice rescued net AHN and ameliorated the depressive-like behavior and a hippocampal-dependent cognitive deficit [25]. This data was supported by subsequent studies in which both acute and chronic sertraline ameliorated a more extensive battery of depressive-like behaviors in female R6/1 HD mice [22, 32]. These data accord with the basic propositions of the neurogenesis hypothesis of depression, where impaired basal AHN levels coincide with depressive-like behavior and the efficacy of pharmacological antidepressants coincides with restoring AHN. Despite neuron production being central to the neurogenesis hypothesis of depression, whether this involves specific deficits at key regulatory points or simply a net AHN reduction remains unclear. Our findings using the R6/1 model have recently questioned whether the neurogenesis hypothesis of depression applies in the context of HD [32]. Activity of specific serotonin receptor subtypes differentially affects net AHN by targeting proliferation, differentiation and survival [33]. Thus tonic serotonin receptor activity may play a role in the maintenance of basal AHN rates. This notion holds particular importance in light of our recent studies showing an imbalance of the serotonergic system in R6/1 HD mice [21, 22, 34]. Impaired serotonergic function in the hippocampus of R6/1 HD mice could play a causative role in reduced AHN in R6/1 HD mice. However, provision of serotonin appears capable of ameliorating depressive-like behavior in R6/1 HD mice without rescuing net AHN levels. Such an observation does not support the neurogenesis hypothesis of depression. 

The yeast artificial chromosome (YAC) mouse model of HD carries a full-length mutant human huntingtin gene with varying CAG repeats lengths [35]. Reflecting the R6 transgenic lines, YAC128 mice (128 CAG repeats) also develop depressive-like behavioral symptoms and hippocampal-dependent cognition deficits [36, 37]. In contrast to the R6/1 study, fluoxetine provided no significant efficacy in redressing depressive-like symptoms in YAC128 mice [38]. Hitherto studies on YAC128 mice have shown that the early onset of depressive-like behavior does not progress in severity with advancing age [37]. Further studies have shown that adult hippocampal neurogenesis deficits in YAC128 mice are progressive and appear to be relatively intact at an age coincident with the manifestation of depressive-like behavior [39]. The progressive deficits in adult hippocampal neurogenesis appear to more closely correlate with the onset of spatial memory and learning impairments in YAC128 mice [36, 37]. Moreover, evidence derived from YAC128 mice does not support the notion that deficits in adult hippocampal neurogenesis precipitate depressive-like behavior, however, these neurogenesis deficits do retain an association with impaired hippocampal-dependent cognition. Finally, a new study has revealed sexually dimorphic affective dysfunction and adult neurogenesis (neuronal maturation but not cell proliferation) deficits in the Hdh^Q111^ knock-in mouse model of HD [40]. Thus, for all of the HD mouse models investigated AHN is abnormal, suggesting that this is a robust effect of the HD gene mutation expressed in different contexts.

Physical activity is advocated to maintain mental health including the prevention and amelioration of depression and anxiety [38]. Physical activity was one of the first physiological factors found to both increase AHN and improve hippocampal function in rodents [41]. The pertinent question arises: does AHN mediate the anti-depressant effects of exercise? It appears that increases in the proliferative steps of AHN are associated with the antidepressant effects of exercise [42]. However, while exercise alleviates depressive-like behavior in R6/1 HD mice, it does not concomitantly elevate neural stem/precursor cell proliferation in the hippocampus [32]. Furthermore, environmental enrichment, which delays onset of affective, cognitive and motor deficits in R6/1 HD mice [19, 21, 43], has only subtle effects on AHN in these mice [27]. Thus, redressing AHN impairment is not necessary for antidepressant effects in R6/1 HD mice. However, part of the neurogenesis hypothesis of depression posits that reduced basal rates of AHN precipitates the development of depression. The early observed lower rates of proliferation of neural stem/precursor cells in R6/1 HD mice [26] are not reflected in a recent postmortem analysis in HD patients [44]. One caveat of this human postmortem finding, using immunohistochemical analysis of endogenous cell-cycle markers, is that the patients who had eventually died from HD (after one or more decades of disease progression) are at a much later stage of progression than was examined in HD mice, and may also have received treatments, such as SSRIs, that are known to affect AHN. Furthermore, AHN is known to decline with age and in this postmortem study [44], where the average age of controls and HD subjects at death was close to 60 years, the baseline cell proliferation detected in the dentate gyrus appeared to be low. Thus, a “floor effect” could mean that any early HD-induced AHN deficit would decrease over time due to age-dependent declining control levels of AHN.

Further consideration relevant to the apparent discrepancies between mouse models and the human postmortem study resides in the observation that the transgenic and knock-in lines have CAG repeat expansions that are in excess of the pathological repeats observed in most patients (although juvenile-onset HD patients do generally have >70 CAG repeats). Nevertheless, the R6/1 and YAC mouse lines show strong construct and face validity, with adult-onset symptoms and molecular abnormalities that do model clinical HD. Furthermore, there is evidence of neuronal cell loss in the hippocampus of HD patients at postmortem [45] as well as hippocampal volumetric evidence from MRI studies [46]. The issue of whether AHN deficits occur in clinical HD will only be definitely answered with new brain imaging approaches which are sensitive to *in vivo* changes in neurogenesis and can be performed longitudinally on gene-positive subjects.

Another molecular correlate of pathogenesis in the hippocampus of R6/1 HD mice, which may contribute to AHN deficits as well as affective and cognitive abnormalities, is decreased brain-derived neurotrophic factor (BDNF) [20, 47, 48]. BDNF is a potent stimulator of adult neurogenesis [49, 50]. In particular, BDNF has been associated with hippocampal synaptic plasticity and neurogenesis and implicated in enhanced hippocampal-dependent cognition. Further evidence for a pathogenic role of BDNF dysregulation in HD has been provided by other animal and cell models of HD, as well as other neurodegenerative diseases (reviewed by [51]). Thus, along with the serotonergic dysregulation discussed above, BDNF and its associated signaling pathways (including the TrkB receptor) are likely candidates to help explain the AHN deficits in HD mice. It is possible that BDNF dysregulation could contribute to affective and cognitive deficits independently of effects on AHN, although this has yet to be experimentally tested. Enhanced voluntary exercise and environmental enrichment were found to differentially affect BDNF expression in the hippocampus of R6/1 HD mice and wild-type controls, which may provide insight into molecular mechanisms mediating the beneficial effects of such environmental manipulations [20, 47, 48]. Other evidence linking BDNF to the beneficial effects of environmental enrichment and exercise on AHN and cognition, particularly with respect to “pattern separation” during memory formation, has been recently discussed [52]. These and other findings have led to BDNF being considered a therapeutic target for various psychiatric and neurological disorders [53]. 

Our present data in R6/1 HD mice suggest that despite reduced net rates of AHN there is no change in proliferation at an age coincident with the presence of depressive-like behavior (Ransome and Hannan, submitted). Collectively, these data are consistent with the possibility that specific deficits in hippocampal proliferation contribute to a subset of cognitive deficits and/or depressive-like behaviors, which can be alleviated through restoring proliferation levels. In the case of R6/1 HD mice, basal proliferation is normal and, therefore, the development of concomitant depressive-like endophenotypes is presumably elicited from other pathologies. This notion is strengthened by the observation that exercise in R6/1 HD mice restores 5-HT1A receptor function thus explaining the amelioration of depressive-like behavior in the absence of AHN effects [32] (Ransome and Hannan, submitted). 

## 3. Clinical Significance 

What is the relevance for HD patients? The collective data using environmental and pharmacological interventions suggest basal serotonergic dysfunction in the hippocampus plays a causative role in depressive-like behavior in R6/1 HD mice. Efforts to ameliorate depression should focus on this system, and perhaps others such as catecholamine and neurotrophin signaling, rather than AHN per se. Another feature to emerge from these recent studies is the evidence for sexual-dimorphism in the development of major depressive disorder. Clinical depression has a higher incidence in females compared to males [54, 55], which intuitively suggests sex-hormone involvement. Much interest continues to surround differential regulation of AHN dependent on sex [56]. Recent studies have highlighted sex-specific AHN increases in response to fluoxetine treatment [57]. Could the neurogenesis hypothesis of depression manifest differently in males and females? Our endeavors to elucidate the role of AHN in the etiology of psychiatric illness have demonstrated that rates of proliferation, differentiation, and survival of adult hippocampal neurons are similar between male and female mice [58]. Furthermore, our current work on R6/1 HD mice shows no change between male and female hippocampal stem/precursors cell proliferation (Ransome and Hannan, submitted). This is reflected in YAC128 mice, in which impaired adult hippocampal neurogenesis manifests similarly in both males and females [39]. The decrease in hippocampal serotonin levels in R6/1 HD mice is more severe in females compared to males, while males respond less to sertraline treatment [22]. Moreover, these sexually dimorphic observations in R6/1 HD mice are consistent with the hypothesis that serotonergic dysfunction (and possibly changes in other molecular systems such as catecholamine and neurotrophin signaling) rather than AHN deficits are central to the manifestation of depressive-like behavior. Again, YAC128 HD mice show a contrasting result, whereby the depressive phenotype manifests equally in males and females [37].

With respect to the link between AHN and cognition in rodents, there are clinical studies in which similar memory deficits have been identified in HD patients, for example, a recent study involving a human analog of the Morris water maze [59]. The clinical incidence of cognitive dysfunction in HD appears to largely manifest equally in male and female patients, although most studies do not assess for potential sex effects. In contrast, it would be expected, based on depression studies in the broader population, that depression would be more common in females than males in those with the HD gene mutation. Surprisingly, this question has not been systematically addressed and is worthy of investigation.

Late-onset hypogonadism is posited to be a readily treatable predisposing factor to depression in elderly men [60]. Clinical analysis shows that male HD patients have lower testosterone levels [61]. This study determined that testosterone levels inversely correlated with the severity of the manifest cognitive impairment but not depressive-like symptoms in these male HD patients. We recently explored the potential of testosterone therapy for cognitive impairment in HD using R6/1 HD mice. Chronic testosterone at supraphysiological doses provided efficacy in restoring testosterone levels, androgen receptor expression, and testicular function in R6/1 HD male mice [62]. However, testosterone therapy did not induce AHN nor rescue cognitive impairment. Androgens can exert anxiolytic effects in mice. Given that our study showed AHN deficits were refractory to the stimulating effects of testosterone in R6/1 HD mice, this model may provide a valuable tool in elucidating the mechanism of testosterone's antidepressant effects including an obligatory role of AHN. This would provide additional evidence toward refining (or refuting) the neurogenesis hypothesis of depression, should testosterone therapy prove to reverse the depressive-like symptoms. 

## 4. Concluding Remarks 

The discovery and characterisation of adult neurogenesis in the mammalian hippocampus has elicited several hypotheses regarding its physiological roles, including contributions to neural systems mediating specific cognitive and affective functions. Accumulating evidence suggests that AHN contributes to specific cognitive processes such as spatial pattern separation [17, 63]. The discovery that pharmacological antidepressants stimulate hippocampal neurogenesis, which in turn appeared to be necessary for therapeutic efficacy of such drugs, provided a significant impetus for the generation of the neurogenesis hypothesis of depression [64, 65]. Accumulating evidence has seen refinements of the hypothesis, where depression manifests at least partly from hippocampal dysfunction precipitated by neurogenesis deficits [66]. Nevertheless, the hypothesis remains controversial and requires further testing in validated animal models. On balance, our data derived from R6/1 HD mice suggest that depression symptoms associated with HD are more consistent with the serotonergic vulnerability hypothesis of depressive disorders [67], although other molecular abnormalities, such as disrupted neurotrophin and catecholamine signaling, may also be involved. 

The potential role of AHN deficits in the pathogenesis of cognitive and affective symptoms in HD has also not yet been fully tested in animal models. Transgenic animal models, such as HD mice, provide a unique system in which the progressive development of affective, cognitive, and motor deficits can be delineated over time, and the preceding molecular and cellular changes can be closely correlated with onset of specific endophenotypes. Similarly, environmental and pharmacological inventions which induce affective and cognitive benefits in HD mouse models can be used to explore molecular and cellular mechanisms of pathogenesis. The psychiatric and cognitive symptoms of HD are amongst the earliest and most devastating in this currently incurable disease. While adult neurogenesis deficits may contribute, other cellular processes, such as synaptic function and plasticity, are known to be disrupted in HD mouse models (e.g., [18, 68–70]) and are likely to be involved in the generation of specific psychiatric and cognitive endophenotypes. By tackling this unique disease with validated animal models that integrate pathogenic processes at molecular, cellular and systems levels, we hope to attain more sophisticated understanding of the complex etiologies involved and thus develop effective therapeutic approaches for HD and related disorders.

## References

[1] Jones EG (1994). The neuron doctrine 1891. *Journal of the History of the Neurosciences*.

[2] Berlucchi G, Buchtel HA (2009). Neuronal plasticity: historical roots and evolution of meaning. *Experimental Brain Research*.

[3] Altman J (1962). Are new neurons formed in the brains of adult mammals?. *Science*.

[4] Altman J, Das GD (1965). Autoradiographic and histological evidence of postnatal hippocampal neurogenesis in rats. *Journal of Comparative Neurology*.

[5] Gross CG (2000). Neurogenesis in the adult brain: death of a dogma. *Nature Reviews Neuroscience*.

[6] Marchetto MCN, Winner B, Gage FH (2010). Pluripotent stem cells in neurodegenerative and neurodevelopmental diseases. *Human Molecular Genetics*.

[7] Gil-Mohapel J, Simpson JM, Ghilan M, Christie BR (2011). Neurogenesis in Huntington’s disease: can studying adult neurogenesis lead to the development of new therapeutic strategies?. *Brain Research*.

[8] Squire LR (1992). Memory and the hippocampus: a synthesis from findings with rats, monkeys, and humans. *Psychological Review*.

[9] Nestler EJ, Barrot M, DiLeone RJ, Eisch AJ, Gold SJ, Monteggia LM (2002). Neurobiology of depression. *Neuron*.

[10] Pittenger C, Duman RS (2008). Stress, depression, and neuroplasticity: a convergence of mechanisms. *Neuropsychopharmacology*.

[11] Phillips W, Shannon KM, Barker RA (2008). The current clinical management of Huntington’s disease. *Movement Disorders*.

[12] Eriksson PS, Perfilieva E, Björk-Eriksson T (1998). Neurogenesis in the adult human hippocampus. *Nature Medicine*.

[13] Lucassen PJ, Meerlo P, Naylor AS (2010). Regulation of adult neurogenesis by stress, sleep disruption, exercise and inflammation: implications for depression and antidepressant action. *European Neuropsychopharmacology*.

[14] Drew MR, Hen R (2007). Adult hippocampal neurogenesis as target for the treatment of depression. *CNS and Neurological Disorders Drug Targets*.

[15] Duman RS, Malberg J, Thome J (1999). Neural plasticity to stress and antidepressant treatment. *Biological Psychiatry*.

[16] Jacobs BL, van Praag H, Gage FH (2000). Adult brain neurogenesis and psychiatry: a novel theory of depression. *Molecular Psychiatry*.

[17] Deng W, Aimone JB, Gage FH (2010). New neurons and new memories: how does adult hippocampal neurogenesis affect learning and memory?. *Nature Reviews Neuroscience*.

[18] Murphy KPSJ, Carter RJ, Lione LA (2000). Abnormal synaptic plasticity and impaired spatial cognition in mice transgenic for exon 1 of the human Huntington’s disease mutation. *Neuroscience*.

[19] Nithianantharajah J, Barkus C, Murphy M, Hannan AJ (2008). Gene-environment interactions modulating cognitive function and molecular correlates of synaptic plasticity in Huntington’s disease transgenic mice. *Neurobiology of Disease*.

[20] Pang TYC, Stam NC, Nithianantharajah J, Howard ML, Hannan AJ (2006). Differential effects of voluntary physical exercise on behavioral and brain-derived neurotrophic factor expression deficits in huntington’s disease transgenic mice. *Neuroscience*.

[21] Pang TYC, Du X, Zajac MS, Howard ML, Hannan AJ (2009). Altered serotonin receptor expression is associated with depression-related behavior in the R6/1 transgenic mouse model of Huntington’s disease. *Human Molecular Genetics*.

[22] Renoir T, Zajac MS, Du X (2011). Sexually dimorphic serotonergic dysfunction in a mouse model of Huntington’s disease and depression. *PLoS ONE*.

[23] Mandrioli R, Mercolini L, Saracino MA, Raggi MA (2012). Selective serotonin reuptake inhibitors (SSRIs): therapeutic drug monitoring and pharmacological interactions. *Current Medicinal Chemistry*.

[24] Rocha FL, Fuzikawa C, Riera R, Hara C (2012). Combination of antidepressants in the treatment of major depressive disorder: a systematic review and meta-analysis. *Journal of Clinical Psychopharmacology*.

[25] Grote HE, Bull ND, Howard ML (2005). Cognitive disorders and neurogenesis deficits in Huntington’s disease mice are rescued by fluoxetine. *European Journal of Neuroscience*.

[26] Lazic SE, Grote H, Armstrong RJE (2004). Decreased hippocampal cell proliferation in R6/I Huntington’s mice. *NeuroReport*.

[27] Lazic SE, Grote HE, Blakemore C (2006). Neurogenesis in the R6/1 transgenic mouse model of Huntington’s disease: effects of environmental enrichment. *European Journal of Neuroscience*.

[28] Walker TL, Turnbull GW, Mackay EW, Hannan AJ, Bartlett PF (2011). The latent stem cell population is retained in the hippocampus of transgenic Huntington’s disease mice but not wild-type mice. *PLoS ONE*.

[29] Gil JMAC, Mohapel P, Araújo IM (2005). Reduced hippocampal neurogenesis in R6/2 transgenic Huntington’s disease mice. *Neurobiology of Disease*.

[30] Phillips W, Morton AJ, Barker RA (2005). Abnormalities of neurogenesis in the R6/2 mouse model of Huntington’s disease are attributable to the *in vivo* microenvironment. *Neuroscience*.

[31] Peng Q, Masuda N, Jiang M (2008). The antidepressant sertraline improves the phenotype, promotes neurogenesis and increases BDNF levels in the R6/2 Huntington’s disease mouse model. *Experimental Neurology*.

[32] Renoir T, Pang TYC, Zajac M (2012). Treatment of depressive-like behaviour in Huntington's disease mice by chronic sertraline and exercise. *British Journal of Pharmacology*.

[33] Klempin F, Babu H, Tonelli DDP, Alarcon E, Fabel K, Kempermann G (2010). Oppositional effects of serotonin receptors 5-HT1a, 2, and 2c in the regulation of adult hippocampal neurogenesis. *Frontiers in Molecular Neuroscience*.

[34] Renoir T, Chevarin C, Lanfumey-Mongredien L, Hannan AJ (2011). Effect of enhanced voluntary physical exercise on brain levels of monoamines in Huntington disease mice. *PLoS Currents*.

[35] Hodgson JG, Agopyan N, Gutekunst CA (1999). A YAC mouse model for Huntington’s disease with full-length mutant huntingtin, cytoplasmic toxicity, and selective striatal neurodegeneration. *Neuron*.

[36] van Raamsdonk JM, Pearson J, Slow EJ, Hossain SM, Leavitt BR, Hayden MR (2005). Cognitive dysfunction precedes neuropathology and motor abnormalities in the YAC128 mouse model of Huntington’s disease. *Neuroscience*.

[37] Pouladi MA, Graham RK, Karasinska JM (2009). Prevention of depressive behaviour in the YAC128 mouse model of Huntington disease by mutation at residue 586 of huntingtin. *Brain*.

[38] Ernst C, Olson AK, Pinel JPJ, Lam RW, Christie BR (2006). Antidepressant effects of exercise: evidence for an adult-neurogenesis hypothesis?. *Journal of Psychiatry and Neuroscience*.

[39] Simpson JM, Gil-Mohapel J, Pouladi MA (2011). Altered adult hippocampal neurogenesis in the YAC128 transgenic mouse model of Huntington disease. *Neurobiology of Disease*.

[40] Orvoen S, Pla P, Gardier AM, Saudou F, David DJ (2012). Huntington's disease knock-in male mice show specific anxiety-like behaviour and altered neuronal maturation. *Neuroscience Letters*.

[41] van Praag H, Christie BR, Sejnowski TJ, Gage FH (1999). Running enhances neurogenesis, learning, and long-term potentiation in mice. *Proceedings of the National Academy of Sciences of the United States of America*.

[42] Bjørnebekk A, Mathé AA, Brené S (2005). The antidepressant effect of running is associated with increased hippocampal cell proliferation. *International Journal of Neuropsychopharmacology*.

[43] van Dellen A, Blakemore C, Deacon R, York D, Hannan AJ (2000). Delaying the onset of Huntington’s in mice. *Nature*.

[44] Low VF, Dragunow M, Tippett LJ, Faull RLM, Curtis MA (2011). No change in progenitor cell proliferation in the hippocampus in Huntington's disease. *Neuroscience*.

[45] Spargo E, Everall IP, Lantos PL (1993). Neuronal loss in the hippocampus in Huntington’s disease: a comparison with HIV infection. *Journal of Neurology Neurosurgery and Psychiatry*.

[46] Bohanna I, Georgiou-Karistianis N, Hannan AJ, Egan GF (2008). Magnetic resonance imaging as an approach towards identifying neuropathological biomarkers for Huntington’s disease. *Brain Research Reviews*.

[47] Spires TL, Grote HE, Garry S (2004). Dendritic spine pathology and deficits in experience-dependent dendritic plasticity in R6/1 Huntington’s disease transgenic mice. *European Journal of Neuroscience*.

[48] Zajac MS, Pang TYC, Wong N (2010). Wheel running and environmental enrichment differentially modify exon-specific BDNF expression in the hippocampus of wild-type and pre-motor symptomatic male and female Huntington’s disease mice. *Hippocampus*.

[49] Zigova T, Pencea V, Wiegand SJ, Luskin MB (1998). Intraventricular administration of BDNF increases the number of newly generated neurons in the adult olfactory bulb. *Molecular and Cellular Neurosciences*.

[50] Benraiss A, Chmielnicki E, Lerner K, Roh D, Goldman SA (2001). Adenoviral brain-derived neurotrophic factor induces both neostriatal and olfactory neuronal recruitment from endogenous progenitor cells in the adult forebrain. *Neuroscience*.

[51] Zuccato C, Cattaneo E (2009). Brain-derived neurotrophic factor in neurodegenerative diseases. *Nature Reviews Neurology*.

[52] Bekinschtein P, Oomen CA, Saksida LM, Bussey TJ (2011). Effects of environmental enrichment and voluntary exercise on neurogenesis, learning and memory, and pattern separation: BDNF as a critical variable?. *Seminars in Cell and Developmental Biology*.

[53] Nagahara AH, Tuszynski MH (2011). Potential therapeutic uses of BDNF in neurological and psychiatric disorders. *Nature Reviews Drug Discovery*.

[54] Grigoriadis S, Robinson GE (2007). Gender issues in depression. *Annals of Clinical Psychiatry*.

[55] Marcus SM, Young EA, Kerber KB (2005). Gender differences in depression: findings from the STAR∗D study. *Journal of Affective Disorders*.

[56] Galea LAM (2008). Gonadal hormone modulation of neurogenesis in the dentate gyrus of adult male and female rodents. *Brain Research Reviews*.

[57] Hodes GE, Hill-Smith TE, Suckow RF, Cooper TB, Lucki I (2010). Sex-specific effects of chronic fluoxetine treatment on neuroplasticity and pharmacokinetics in mice. *Journal of Pharmacology and Experimental Therapeutics*.

[58] Manning EE, Ransome MI, Burrows EL, Hannan AJ (2012). Increased adult hippocampal neurogenesis and abnormal migration of adult-born granule neurons is associated with hippocampal-specific cognitive deficits in phospholipase C-*β*1 knockout mice. *Hippocampus*.

[59] Majerová V, Kalinčík T, Laczó J (2012). Disturbance of real space navigation in moderately advanced but not in early Huntington's disease. *Journal of the Neurological Sciences*.

[60] Almeida OP, Yeap BB, Hankey GJ, Jamrozik K, Flicker L (2008). Low free testosterone concentration as a potentially treatable cause of depressive symptoms in older men. *Archives of General Psychiatry*.

[61] Markianos M, Panas M, Kalfakis N, Vassilopoulos D (2005). Plasma testosterone in male patients with Huntington’s disease: relations to severity of illness and dementia. *Annals of Neurology*.

[62] Hannan AJ, Ransome MI (2012). Deficits in spermatogenesis but not neurogenesis are alleviated by chronic testosterone therapy in R6/1 Huntington's disease mice. *Journal of Neuroendocrinology*.

[63] Clelland CD, Choi M, Romberg C (2009). A functional role for adult hippocampal neurogenesis in spatial pattern separation. *Science*.

[64] Malberg JE, Eisch AJ, Nestler EJ, Duman RS (2000). Chronic antidepressant treatment increases neurogenesis in adult rat hippocampus. *Neuroscience*.

[65] Santarelli L, Saxe M, Gross C (2003). Requirement of hippocampal neurogenesis for the behavioral effects of antidepressants. *Science*.

[66] Kempermann G, Krebs J, Fabel K (2008). The contribution of failing adult hippocampal neurogenesis to psychiatric disorders. *Current Opinion in Psychiatry*.

[67] Jans LAW, Riedel WJ, Markus CR, Blokland A (2007). Serotonergic vulnerability and depression: assumptions, experimental evidence and implications. *Molecular Psychiatry*.

[68] Milnerwood AJ, Raymond LA (2010). Early synaptic pathophysiology in neurodegeneration: insights from Huntington’s disease. *Trends in Neurosciences*.

[69] Raymond LA, André VM, Cepeda C, Gladding CM, Milnerwood AJ, Levine MS (2011). Pathophysiology of Huntington's disease: time-dependent alterations in synaptic and receptor function. *Neuroscience*.

[70] Nithianantharajah J, Hannan AJ Dysregulation of synaptic proteins, dendritic spine abnormalities and pathological plasticity of synapses as experience-dependent mediators of cognitive and psychiatric symptoms in Huntington’s disease.

